# Meteorology and British film studios: an article of the London fog

**DOI:** 10.1080/01439685.2021.1922033

**Published:** 2021-05-13

**Authors:** Richard Farmer

## Abstract

*Industrial winter fogs posed an almost existential threat to filmmaking in Britain during the first decades of the twentieth century, disrupting outdoor or location filming, penetrating studio buildings and delaying production schedules and increasing costs. The problem was exacerbated by many British studios being located in, or on the outskirts of London, a city famous for its ‘pea soupers’. This article, having outlined the nature and extent of the problem, explores the ways in which the British film industry responded to the fog. Some studios ceased operations in the winter, whilst some producers relocated to less meteorologically-challenging climes. Two other responses, however, allowed for year-round production in Britain: the installation of specially designed fog-dispersal plant, and the construction of new studios outside the London ‘fog-zone.’ Using fog to explore the impact of climate on the siting, design and equipping of British film studios, the article is intended to encourage recognition of the ways in which the spaces and infrastructure of film production develop in relation to specific local, regional or national level factors, and stimulate discussion of the relationship between filmmakers, studios and the natural (or, in the case of the London fog, anthropogenic) environments within which they operate*.

There is nothing in the world quite like a London fog. You breathe black foulness and it enters into you and contaminates you.

Arthur Symons, ‘London: A Book of Aspects’ (1909) in *Cities and Sea-Coasts and Islands* (Evanston, IL: Marlboro Press/Northwestern, 1998 [1918]), 211-12)

The fact that every British kinema studio is now idle … is due, to a very large degree, to the fact that our weather conditions in November and December are inimical to interior photography.

*Westminster Gazette*, 19 November 1924, 6.

The British film production industry, from its very inception, found itself at the mercy of the winter weather. In particular, winter fogs were such a perennial problem that they posed an almost existential threat to the British production sector. For decades, Symons’s ‘black foulness’ – a toxic combination of naturally occurring fog and manmade coal smoke and/or other industrial pollutants – proved as capable of entering into a building as a human body, and had a similarly corrupting effect.^1^ As *Kinematograph Weekly* noted in 1921, ‘there are many studios in and around London into which the fog easily penetrates, and great difficulty is experienced in removing it.’^2^ Once inside a studio, fog reduced visibility, refracted light, and made shooting impossible; it played havoc with both schedules and budgets. In Britain, the fog was so disruptive a presence that, although each individual instance was intangible and ephemeral, it had a formative influence on the siting, design and equipping of film studios. Despite evaporating each year in the spring sunshine, and all but disappearing as a result of the 1956 Clean Air Act, industrial fog had a lasting impact on the material spaces of British film production that can still be seen today. This article will explore fog as a spectral presence within British film history, and will focus predominantly on the British production sector in the 1920s and 1930s, although fog continued to disrupt British film studios into the 1950s.^3^ As such, it speaks to scholarship that has sought to understand the relationship between filmmaking and meteorology, not so much in terms of its thematic presence within films, although that will be discussed, but rather in terms of how geography and climate influenced the location, architecture and technology of film production.^4^

Fog was especially disruptive to the British production sector because that part of the British film industry has, for much of its history, been concentrated in and around London, a city so regularly affected by dense, industrial fogs that its inhabitants coined phrases such as ‘pea souper’ and ‘London particular’ to describe them.^5^ Such fogs became less frequent during the course of the twentieth century, as more homes started to cook with gas hobs rather than coal fires, and as population density was reduced by greater suburbanisation.^6^ However, fogs continued to descend, unannounced and unwelcome, to disrupt life and labour throughout the 1920s and 1930s, and a list of ‘noteworthy’ London fogs includes examples from the 1960s.^7^

The idea that climatic factors have had a formative impact on film history is not new, of course. The notion that the warm, sunny climate of California had a part to play in enticing the fledgling American industry away from the East Coast is a commonplace of film history – even if, as Brian Jacobson has noted, ‘filmmakers hoping for a favourable climate year-round were in for an unpleasant surprise’.^8^ Britain, however, did not offer filmmakers a choice of distinct climatic regions, although the east of the country tends to be drier than the west, and the south milder and sunnier than the north. Consequently, British studios were often products of design processes obliged to keep the disruptive potential of the weather in mind, as this 1936 report on the newly opened Denham makes clear:

the difficulties encountered in designing a studio in England are considerably more than they are in America. Admittedly a great deal has been learnt from that pioneer country in studio construction, but however many tips it has been possible to borrow from California, it has not been possible to import the greatest of her gifts, her golden climate. It is the English weather which has been arch dictator in the design of these studios, by making, as the first essential, the provision of absolute immunity from all its vagaries.^9^

At Denham, the studios were designed to be as compact as possible (occupying 29 acres of a 165-acre site), with covered walkways built between many departments in order to minimise employees’ exposure to the elements.^10^ Similarly, at Pinewood a 15,000 sq. ft. area was covered as a ‘wise provision’ against the climate:^11^ ‘If a director on stage 4 … wants a stag’s head with antlers, a lithograph of Queen Victoria, a pillar box, or an Egyptian mummy case, he can get them without ever emerging into the open.’^12^ Design features such as these relate more to rain than fog, but nonetheless make clear that the weather was a constant companion for filmmakers in Britain, and one that shaped the spaces in which they worked.

Indeed, so important was climatic data to the planning and design of British film studios, that the British government’s Meteorological Office made ‘special mention’ of the numerous enquiries it fielded on the subject.^13^ The need for studio designers, both within and outwith Britain, to mitigate local climatic conditions by means of siting and technology brought about the construction of what might be termed aclimatic filmmaking environments. Such studios were designed to create interior spaces that could operate independently of external meteorological events. However, as far as studio filmmaking was concerned, not all weather was created equal, and many British producers found to their cost that fog was a particularly insidious adversary.

## Impact on production

With much early filmmaking taking place outdoors, or in studio spaces partially or wholly reliant on sunlight for illumination, adverse weather conditions had considerable disruptive potential. Although a British summer offered no guarantees of good weather – Edison’s Charles Brabin lost approximately half of shooting days on a trip to Britain from May to October 1913^14^ – filmmakers discovered that attempting to make films in the late autumn and winter was especially risky. Shorter daylight hours, weaker sunlight, greater chances of overcast or wet weather, and the advent of the annual fog season, which usually began in earnest in November and lasted until March or April, made filming much more challenging, and at times impossible.^15^

Given that they continued to be shot on location, even after the production of fiction films had moved into the studio, topical or actuality films proved to be particular hostages to meteorological fortune. However, filmmakers shooting on location found that different fogs posed different problems: white mists comprised almost entirely of water vapour were far less disruptive than ‘yellow’ urban fogs which had ‘a powerful effect in cutting off light from the scene photographed’.^16^ When Will Barker introduced *London Day by Day* in July 1906, for example, he found the summer weather conducive to the successful production of a daily news film. Come the winter, however, London’s ‘pea-soup fogs’ made it ‘impossible to take fresh pictures every day’ and Barker was forced to bring the series to a premature halt.^17^

Barker would continue to gamble on the weather, and in winter 1910-11 his attempt to film Herbert Beerbohm Tree’s acclaimed stage version of *Henry VIII* was frustrated by the climate on more than one occasion. Barker’s studio at Ealing, a ‘miniature Crystal Palace,’^18^ used the sun as its primary source of illumination, meaning that when Tree and his company arrived at the studio on the morning of 17 November to find the studio engulfed in a thick fog, there was insufficient light for filming. Tree left Ealing without a single foot of film being exposed.^19^ A report in the *Daily Telegraph* suggested that this situation ‘brought most annoyance’ to Tree and his players, but Barker would surely have been similarly frustrated: he had spent approximately £150 on pantechnicons to bring costumes and props from His Majesty’s theatre, only to return them unused. Another attempt was made on 22 November, but the weather ‘was again unpropitious,’ and it was not until 9 February 1911 that *Henry VIII* was finally committed to celluloid.^20^ The film’s critical and commercial success suggests that the delay caused by the fog did not damage its prospects, even if it had increased the chances that the film might not capitalise on the excitement surrounding Tree’s theatrical production, which had opened in September. Nevertheless, Barker had made a significant investment in building and constructing ornate sets for the film and storing these and watching them sit idle would have constituted a financial burden.

Fog was a persistent seasonal problem because the British production sector remained undercapitalised until at least the mid-1920s. In both purpose-built studios and those converted from existing buildings, many ‘essentials appear to have been scamped’^21^ and design features that might have prevented fog ‘pouring in at every chink and keyhole,’ as Dickens put it in *A Christmas Carol,* were either not installed, or installed ineffectively. Consequently, many British studios were, as one observer noted in 1925, ‘practically useless in the winter’.^22^ Producers were encouraged to seal window frames and doors more effectively, but the obvious need for technicians and actors to enter and exit stages could undermine the efficacy of such work; installing air-traps (two sets of doors, with only one opened at any given time) was proposed as a possible solution, although this was more expensive to implement and, if retro-fitted, could reduce a stage’s usable space.^23^ Even after a studio had installed equipment to counteract problems caused by fog, open doors were capable of causing significant disruption. The production of *Love, Life and Laughter* (1923) ran into problems when a particularly deep set, built on the No. 1 stage at Islington, required that the camera be placed outside the open studio doors, thereby admitting a ‘thin but deadly’ fog: ‘for an hour or more at a time, everyone from Betty Balfour … downwards, would be held inactive while mammoth air-filtering machinery ran a race with the incoming current through that open door where more and more fog kept streaming through to replace what was pumped out’.^24^

Weather-related production delays came at a cost. In February 1921, fog was said by *Kinematograph Weekly* to have had a ‘disastrous effect’ on many production companies working in London:

Two companies at least had to suspend work and many other productions were delayed by the dull, foggy weather. The result was that hundreds of pounds were wasted on lights, supers [i.e. extras], and other details.^25^

If anything, the early part of the winter of 1923-4 was even worse. Producer George Cooper reported that during the course of eighteen working days in December, ‘the camera had turned for no more than thirty-two hours.’^26^ When the release of Cooper’s *Claude Duval* (1924) was held up by eight months, he blamed ‘the terrible weather conditions of the late unlamented winter.’^27^ Even allowing for a fair degree of hyperbole, the weather evidently posed a very serious problem, not least because desperation to make up for lost time resulted in Cooper turning the cameras before the fog had sufficiently dissipated, necessitating a ‘large percentage’ of additional retakes, and further expense. All in all, Cooper observed, ‘the worries of directing stars, story and staging, were as nothing compared with the anxiety of helplessly watching the commercial prospects of the picture being day by day more jeopardised by the abominable weather.’^28^

Whilst the additional costs associated with fog were problematic for individual production companies, the winter weather had a profoundly negative impact on the British production sector as a whole. ^29^ As late at the second half of the 1920s, studios operated flat out during late spring, summer and early autumn in order that filming could be completed before the onset of the fog season made shooting a lottery.^30^ The annual mothballing of studios and personnel, which would have reduced but not entirely eliminated overheads, served to undermine the competitiveness of the British industry as compared to rival film-producing nations, especially the United States. What was already a smaller industry found its output further reduced by fog, leaving distributors and exhibitors with little choice but to book films produced outside the United Kingdom. There were, of course, a host of factors that facilitated the domination of British screens by overseas- and especially American-produced films, but until the 1927 Cinematograph Films Act encouraged greater investment in British production facilities, that most stereotypical of British obsessions, the weather, made it more difficult for British filmmakers to put British life on the screen.

## Fog and filming

Although some producers were content for the fog that infiltrated their studios to be perceived as ‘artistic soft-focus photography’, most filmmakers regarded fog as problematic because it hindered vision.^31^ Thick fog was capable of completely obscuring the object that the camera was hoping to photograph, but, as Colin N. Bennett noted in *Kinematograph Weekly* in 1922, even a very thin fog could cause significant disruption: water, a constituent element of fog, reflected more actinic light than it did other parts of the spectrum, rendering it more visible to the orthochromatic film stock commonly used in this period than it was to the human eye.^32^ Panchromatic film stock, which became more widely available during the 1920s, was sensitive to a wider part of the spectrum and so reduced the severity of such problems. Indeed, when panchromatic stock was used in conjunction with a screen or colour filter, it became possible to ‘cut out haze which is visible to the eye.’^33^

The progression from outdoor stages to sunlit steel-and-glass studios, to ‘dark,’ artificially illuminated facilities in the first decades of the twentieth century provided a means by which natural light lost to cloud, dusk, the changing season or fog could be supplemented or eventually replaced by electric alternatives. Increasingly advanced studios created ever ‘more manageable … environments’ capable of ‘eliminating rain, wind, snow, heat and cold.’^34^ However, British filmmakers discovered that blacking out windows and constructing a water-fast roof did not necessarily guard against fog, which proved to be a persistent opponent, and which could be as disruptive of artificial lighting as it was of sunlight. Artificial lights presented an additional problem: even thin mists became visible under the fierce glare of the lamps. As one British cameraman put it in 1921, ‘you may “shoot” in the dark by means of light, but you cannot “shoot” in a fog no matter how powerful your lights may be.’^35^ The truth of this was proved in 1923, when the producers of *The Money Habit* (1924) found that the stage at Islington was ‘so thick [with fog] that “taking” was impossible’ during the day, and so decided to shoot from 9 pm until 5 o’clock in the morning.^36^

Atmospheric conditions were also believed to have an effect on studio acoustics, a problem that became more immediate following the advent of synchronous sound. Writing in the *Bioscope*, Kenneth J. Robertson advanced the idea that just as they did in outdoor locations, internal atmospheric conditions altered the transmission and recording of sound, and observed that the presence of fog in a sound stage could bring about a ‘muffled stillness.’^37^ Elsewhere, however, it was suggested that the coming of the talkies might actually benefit producers in their struggle against the fog. The limited range and pick-up of early studio microphones encouraged a greater use of close shots, reducing the amount of fog that the camera had to penetrate before capturing an actor’s image.^38^ Further, acoustic insulation installed as part of the sound-proofing process provided for a more rigid separation of interior from exterior spaces, an additional consequence of which was that it became harder for fog to enter the studio.^39^

Moreover, the vaporised carbon produced by the arc lamps used in many silent-era studios was liable to be picked up as haze by the camera, but the noise made by such lamps meant that they were often replaced by tungsten incandescent lights when studios converted to sound. The light produced by these new lamps tended towards the red end of the spectrum, and as orthochromatic stock was not sensitive to red light, the use of incandescent lamps hastened the more widespread use of panchromatic film, an additional benefit of which was that it could more readily counteract the visibility of fog.^40^

The relationship between fog and the technological apparatus of film production was, therefore, multifaceted and dynamic. As filmmaking technologies changed, and as the spaces of film production evolved, so too did the ways in which filmmakers sought to counteract fog. It remained the case, though, that prevention was better than cure: developing fog-free studios was clearly preferable to developing ways to work around the problem, permitting both cleaner, sharper images and greater consistency between shots.

## Trying to solve the problem: a range of options

Presented with persistently poor winter weather, British filmmakers could choose between a number of different options. They could do nothing; either accepting that they would lose time and money to fog and other poor climatic conditions (essentially gambling that these losses would be smaller than the costs associated with pursuing one of the more capital-intensive solutions), or deciding to shut down production during the winter.^41^ A more pro-active option was for filmmakers to transfer production to places where the weather was more conducive to winter filmmaking.^42^ Whilst this allowed individual films to progress, it left British studio facilities and technicians periodically un- or under-employed, even though on many occasions British actors and cameramen travelled with productions rather than such personnel being hired locally.^43^ To keep British facilities operational on a year-round basis, however, studios could either install equipment to combat the fog, or relocate to sites outside the London fog zone. These solutions were likely to be more expensive in terms of up-front costs, but promised to repay initial investment by allowing filmmaking to continue throughout the winter. Each of these responses to the fog was deployed, with the former two becoming less common as investment in the latter two bore fruit, especially after the passing of the Cinematograph Films Act in 1927 promised greater financial stability for the British film industry whilst making it substantially less attractive to make British films in overseas studios.

The First World War put a stop to British commercial filmmakers travelling to continental Europe to make films, but following the Armistice they quickly made up for lost time; Rachael Low identified the years after 1918 as a ‘golden age’ for location shooting in continental Europe, not least because of the ‘better [winter] weather’ offered in France, Spain and Italy.^44^ Indeed, one observer claimed, perhaps facetiously seeking a silver lining to this particular low-lying cloud, that ‘the proximity of London to the Continent’ was one of the city’s ‘chief advantages’ as far as film production was concerned, reducing the cost and logistical difficulties of relocating to mainland Europe for the winter.^45^ Whilst some British filmmakers took advantage of local scenery, or set films either partly or wholly in continental locations, others preferred to simply make use of the more consistent winter sunlight and the absence of fog to shoot purportedly ‘British’ scenes. Exteriors for *The Persistent Lovers* (1922) were shot in England before the onset of ‘fogs and inclement weather,’ whereas interiors were shot after ‘staff, equipment and company’ – more than twenty people in all – had decamped to the south of France having ‘acquired studios on the Riviera for the winter months’.^46^

The Stoll studio in Cricklewood, north London, was regularly affected by winter weather, and one incursion of fog was reported to have ‘got into the studio for about three months’.^47^ The company responded by moving production of films such as *The Prodigal Son* (1923) to Nice. Maurice Elvey, who worked for Stoll in the early 1920s, became convinced that ‘British pictures can be produced abroad better than they can be in Britain’ because ‘for seven months in the year producing in Britain is so handicapped by the climate that it does not give the producer a fair chance.’^48^ Elvey was enthusiastic about the facilities and climate on the French Riviera, and made numerous films in Nice. Although British filmmakers would continue to film overseas during the 1930s, and would resume the practice in the 1940s, fewer were compelled to do so by poor winter weather affecting domestic production.^49^

Escaping the London fog did not necessarily require British filmmakers to travel abroad, and producers instead sought to find locations for studios outside the London fog-zone. Although numerous factors encouraged the development of new studios in rural and semi-rural locations around London – perhaps the most significant of which was lower land prices – the importance of the weather, and fog in particular, was a key consideration. However, the spread of London’s suburbs meant that facilities built in areas supposedly less prone to urban, anthropogenic fogs could find themselves surrounded by the city and its pollution: a studio such as Ealing, constructed in a semi-rural location, was enveloped by London as it expanded inexorably into the surrounding countryside.

The desire to avoid London’s fogs might have inspired the development of the ‘Brighton school’ of British film pioneers, but the gravitational pull of the capital, at the centre of Britain’s transport infrastructure and with its established entertainment industry and millions of ready consumers, persuaded some producers to construct studios on sites close to the city but – they hoped – outside its fog-zone.^50^ The belief that Ealing experienced the greatest ‘freedom from fog of any district within the metropolitan area’ persuaded first Will Barker and then Basil Dean that it was a suitable location for a studio, although, as we have seen, the production of *Henry VIII* demonstrated that the locale was far from fog-free.^51^ When British Instructional opened a studio at Welwyn in 1928, potential customers were informed that the location was blessed with ‘a remarkable clarity of atmosphere’ because ‘chemical fogs are almost unknown.’^52^ Whilst that ‘almost’ might have given producers pause for thought – as might the fact that British Instructional saw fit to install ‘thoroughly efficient’ fog-dispersal plant so as to permit ‘the continuous production of films even during the worst foggy weather’^53^ – the promotion of pollution- and fog-free air as one of the studio’s attractions speaks volumes about the problems associated with atmospheric pollution.

London’s position in a low-lying basin, hemmed in by hills, exacerbated its propensity to fogginess; colder air, and the pollutants it contained, could get trapped underneath a layer of warmer air that had risen above the hills, a phenomenon known as a temperature inversion. During some inversions, the more coal that Londoners burned to counteract the cold, the more unpleasant atmospheric conditions became. Building studios on higher ground therefore allowed them to rise above the fog and made such studios more appealing, especially to independent producers. The Welwyn studio was situated some 400 feet above sea level, and Milheath Studios at Bushey enjoyed a similarly elevated position. Indeed, in 1935 a proposed £200,000 expansion of this latter studio was in part justified by the business it was believed might be attracted by the site’s altitude.^54^

Even lower-lying studios sought to highlight their elevation. In 1927, the promoters of a new studio in Wembley announced that the site was 110 feet above sea level and that local atmospheric conditions were ‘vouched for by a full and favourable’ report from the Meteorological Office.^55^ The accuracy of this report might have been called into question in October 1929, however, when thick fog delayed the arrival of firemen at a blaze that destroyed two of the studio’s stages and caused £150,000 damage.^56^ Indeed, 1925 plans to build a national studio complex at Wembley were abandoned in part because the district’s position within the London fog-zone meant that the site might not be usable on a year-round basis.^57^

The westerly direction of London’s prevailing winds resulted in a tendency to construct new studios to the west of the city, thereby avoiding the plume of pollution emitted from London’s domestic and industrial chimneys. As the British production sector matured, even London’s urban studios began to show a more pronounced tendency towards the western side of the city centre. As production facilities became more sophisticated and expensive, and as budgets for individual films rose, the cost of weather-related disruption became greater; few large studios in or to the east of the city survived into the sound era. Major new facilities were built to the west of London at Shepperton, Denham and Pinewood.

The move to sites outside London pre-dated the First World War. Percy Nash’s decision to build the Neptune studio at Elstree, Hertfordshire, was in part dictated by his desire to escape the kind of foggy weather that had often interrupted his work at studios in Twickenham and Walton-on-Thames:

He tried the higher ground on the north of London. At Mill Hill he noticed a factory manufacturing photographic supplies and concluded he was getting out of the fog belt. Farther afield, at Boreham Wood, he found a site and built himself a small red brick studio with one stage.^58^

Over the subsequent decades, other studios would also be constructed at Elstree-Boreham Wood, and these commonly made a point of referring to the town’s position outside the London fog-zone, even if the advantages of the location were sometimes more hoped for than actual. In December 1925 British National Pictures announced its intention to build a studio in Elstree, and noted that the location had been selected ‘after inspection of many other possible sites’ because of ‘its nearness to London, where the materials of film production are most readily available, and because the Meteorological Office has reported most favourably upon its uniform freedom from fogs.’^59^ However, Elstree’s proneness to fog became something of a joke, not least on the occasion of an event held to announce the expansion of Rock (ex-Neptune) studios in 1936, which went ahead in very challenging conditions: ‘it would be interesting to know who started that yarn about Elstree being outside the fog belt!’^60^ When Consolidated Films took over Elstree’s Whitehall Studio in 1934, it attempted to strike a confident note about the local climate: ‘What fog may arise from time to time is so comparatively slight that the original studio builders did not think it worthwhile to install special air-conditioning plant.’ That decision, the new owners noted laconically, ‘is being corrected.’^61^

Although Elstree was evidently not the fog-free idyll producers had hoped, studios based in the town actively sought to keep the air as clean as possible. Whilst naturally occurring fog could not be prevented, the film industry had some control over pollutants that contributed to anthropogenic fog. Erecting studios in a previously semi-rural district acted as catalyst for greater urbanisation, both through the construction of the studio and the building of new homes to accommodate studio employees.^62^ Studio managers were understandably anxious that pollution be kept to a minimum, and might have looked uneasily at a proposal to build 8,000 new homes in Elstree to accommodate as many as 50,000 people.^63^ As such, when the Housing Corporation of Great Britain and Realty Trust Ltd acquired approximately 1,000 acres of land to build an anticipated 3,000 houses in Elstree-Boreham Wood, studio representatives worked with the developers to mitigate the pollution they might cause: it was agreed that only electric lights would be installed and that only smokeless fuel be burned in the grates.^64^ Having escaped London, the film colony was adamant that Elstree ‘has no intention of creating fogs of its own.’^65^

The studios at Elstree were not the only ones built in locations promising cleaner air. George Clark Productions left Ebury Street in Belgravia because proximity to the Thames had ‘the drawback of causing a great loss of time through the heavy atmosphere and fog which occasionally succeeds in entering the studio and holding up the work of production.’^66^ In 1922, Clark moved to a new studio at Beaconsfield in Buckinghamshire, 40 minutes from central London by train but considerably closer to the metropolis than southern France, where he had spent previous winters making films in seasonal exile. At Beaconsfield, the company found not only a location with countryside suited to exteriors, but also climatic conditions that permitted their filming – a Shangri-La where it was possible ‘to work the whole year round and forget that such a thing as fog exists.’^67^ Whilst this enabled Clark to keep working on his own films during the winter, it also allowed him to entice other producers to Beaconsfield: in December 1923, the Granger-Davidson production *Eugene Aram* (1924) transferred ‘owing to foggy conditions’ to Beaconsfield from Walthamstow.^68^ In 1936, an article about the Beaconsfield studio, now under British Lion’s control, claimed that fog remained ‘unknown’ in the area, and that ‘exterior work, winter or summer, has never been held up by this feature of the English weather.’^69^

Because fog is formed when water vapour condenses in cold air, it was possible to prevent fog from forming in the studio by heating the interior of the stages. This also had the effect of evaporating fog that might enter the studio from outside. When Elstree studio was completed in October 1926, the inclusion of an ‘elaborate’ heating system meant that ‘no delay was caused by fog or mist’ during the studio’s first winter.^70^ The nearby Whitehall studio boasted an underfloor heating system − 90 pipes of 2 inch diameter – which prevented fog from interrupting filming.^71^ On occasion, the heating systems used to combat fog created additional problems, as during the filming of *Chu Chin Chow* (1934) at Islington: ‘Lighting from 170 lamps reproducing Eastern [i.e. Asian] sunshine, added to the hot air pumped into the studio in order to keep out fog, developed a heat which compelled the lamp-minders and electricians to work stripped to the buff.’^72^

Inducing evaporation of air-borne water droplets did not necessarily end the problems, though, as industrial fogs comprised condensed water and particles of pollution. Both reflected light, and both could be seen by the camera even if they were not visible to the human eye, so both needed to be eliminated from the shooting stage. However, evaporating water did not rid get rid of pollution, so studios also needed to clean the air that entered into and circulated around spaces in which filming happened. Various solutions were proposed, including the (potentially hazardous) idea of using direct current brush discharge to ‘bring down soot particles’. There is no evidence that this solution was ever implemented, most likely because, as *Kinematograph Weekly* noted, such equipment was ‘not altogether without effect upon human beings in its neighbourhood. Drowsiness and nervous symptoms are believed to be sometimes brought on by it.’^73^

The Famous Players-Lasky studio in Islington was the first in London to install equipment specifically to dispel fog from its production spaces. The studio was situated on the banks of the Regent’s Canal, a location described as ‘the very worst position for fog in the whole of London’.^74^ An American company seeking to open a European subsidiary, Famous Players-Lasky was said to have only ‘partly realised’ the difficulties associated with making films in London’s challenging atmospheric conditions.^75^ It would soon gain a greater understanding of what Jesse Lasky himself decried as the ‘maddening fog’.^76^ When the Islington studio opened in the spring of 1920, high- and low-pressure coils were installed on opposite walls of the stages to circulate foggy air towards the roof, where it was expelled by an exhaust fan. Initial tests found that even when the fog could not be entirely dispersed, it could be raised to a height of 15 feet above the floor, allowing production to continue underneath.^77^

Come the autumn, however, the system was found wanting, even after the decision was taken to reduce the chances of fog entering the studio by sealing the building twenty-four hours before shooting was due to commence.^78^ In October 1920 ‘London was visited by a period of fog [and] and production [in the studio] was held up for no less than a week, because climatic conditions made photography a matter of sheer impossibility.’^79^ During Famous Players-Lasky’s first year at Islington, twenty working days were interrupted by fog,^80^ and the studio’s manager noted that the ‘enforced cessation in our activities … meant to our firm at the lowest estimate a loss of £11,000.’^81^

Consequently, the £7,000 spent attempting to resolve the fog problem made sense. Designed by W. E. Riley, who as the London County Council’s chief architect had helped design the London Underground’s ventilation plant, and S. L. Groom, of the Carrier air-conditioning company, the new system circulated up to 3.5 million cubic feet of air per hour, drawing it from outside the studio before cleansing it of airborne particulates, saturating it, and adjusting its temperature to prevent condensation and ensure a comfortable working environment. So treated, the air was pumped into the studio at a pressure low enough not to raise dust, but ‘slightly greater than the normal outside pressure, and by this means to create a tendency in the atmosphere to leak outwards from the studio’ and so prevent fog from leaking in.^82^ Lasky observed in his memoirs that the use of the equipment occasionally caused ‘inexplicable breezes that caressed the fabric of the heroine’s evening gown in drawing-room scenes.’^83^ To ensure victory over the ‘arch-enemy of good photography,’ the system was automated to react to the changing weather, but also responded as conditions changed inside the studio, such as when temperatures increased when lamps were switched on.^84^

The fog-dispersal plant installed at Islington in time for the winter of 1921-22 had an immediate and beneficial impact on atmospheric conditions inside the studio. In the three years after installation, only two days were lost per year to fog.^85^ Famous Players-Lasky could do nothing to control atmospheric conditions outside the studio, however: exterior shooting continued to be restricted to spring and summer, when the weather was ‘more favourable for location work,’ and a number of the company’s productions travelled to Spain and Italy to conduct location shooting away from the fog, gloom and rain of the British winter.^86^

The fog-dispersal plant at Islington was updated again in 1931-32, based on a system that new owners Gaumont-British had installed at their other studio in Shepherd’s Bush. Indeed, Shepherd’s Bush was almost as prone to fogs as Islington; when the studio was expanded in 1927, Gaumont-British was eager to ensure it was able to operate year-round. Rather than using water to wash the air, the new system extracted impurities by means of fabric filters. This process had been developed by the engineering firm Hall & Kay Ltd. for use in the Lancashire cotton mills, and Gaumont-British first encountered it whilst filming *Hindle Wakes* (1927) on location in Manchester.^87^ Hall & Kay adapted the system for use in a film studio, and the new plant withstood the attentions of a particularly aggressive pea-souper in late November 1927:

On Saturday – the foggiest morning of the year – three of the directors producing in the London area had to stop owing to fog. At Shepherd’s Bush, however, shooting on *Sailors Don’t Care* [1928] was in full swing, and although the outside atmosphere was so thick that 10ft. was about the maximum distance vision, the interior of the studio was absolutely clear.^88^

So effective was the Hall & Kay plant that the company boasted in the *Bioscope* in December 1930 that not a day had been lost to fog since it had been installed. The same advertisement claimed that ‘it has been estimated that on *Quinneys* [1927] alone the entire cost of the outfit was saved’.^89^ So successful was the system that an advertisement for it had to mock up a photograph of a foggy studio for a ‘before’ shot to be contrasted with the pristine, clear air produced by the plant itself.

From the early-1930s, the British trade press carried fewer reports detailing the technical specifications of fog-dispersal plant, suggesting an assumed familiarity with the equipment on the part of its readership. Major new studios built in rural locations, such as those at Denham and Pinewood, installed fog-dispersal plant, and British studios catering to independent producers continued to refer to their possession and use of such equipment, recognising that failure to do so might make them less competitive.^90^ In April 1938, Stoll, a company that in 1927 had boasted of losing ‘*only* seven work-days … to fog’ the previous year, ran an advertisement seeking to entice producers to ‘return to town’ from more recently-built facilities outside London.^91^ Yet clearly concerned about London’s ongoing reputation for fog, the advertisement also foregrounded the measures that had recently been taken to counter the weather; the ‘latest addition’ to the studio’s modern equipment was ‘fog filtering plant’.^92^

The installation of effective fog-dispersal plant, and the construction of new studios at locations outside London’s fog-zone, created and maintained conditions that enabled British film production on a year-round basis. An examination of British trade publications such as *Bioscope* and *Kinematograph Weekly* shows that reports of disruption by fog became fewer and further between as the 1920s and 1930s progressed. Whilst this might be in part attributable to ‘London particulars’ becoming less frequent, it also suggests the success of the measures instituted by British studios to counter the disruption caused by fog.

## Conjuring fog out of clear air

Charlotte Brunsdon has noted that fog functions as a meteorological landmark in many cinematic representations of London, as recognisable a signifier as red double-decker buses or Tower Bridge.^93^ Brunsdon’s focus is on films made after 1945, but fog’s status as a synecdoche for London can be traced back at least as far as the Victorian period, and Peter Ackroyd suggests that the London fog was ‘the greatest character in nineteenth-century fiction’ and, hence, ‘the world’s most famous meteorological phenomenon’.^94^ Fog therefore became essential to filmmakers seeking to create, for both British and international markets, versions of London that accorded with the city as it existed in the cultural imagination. Yet from the birth of cinema into the 1950s, fog was also a pre-requisite for filmmakers seeking to produce a cinematic London that accorded with the city as it was often experienced in reality. The Great Fog of December 1952, for example, inspired its own mini-sequence of films, including *The Runaway Bus* (1954), *Out of the Clouds* (1955) and *Tiger in the Smoke* (1956). Evidently, the desire to keep fog out of the studio did not equate to a desire to keep it out of films.

However, filmmakers, as one journalist learned on a studio visit in 1932, ‘would never use a real fog’ as this risked sullying ‘the purest air in London’.^95^ Rather artificial fog, more biddable, consistent and photogenic than the real thing, was required. It is ironic that a film such as *The Lodger* (1927), subtitled *A Story of the London Fog*, began shooting in March 1926, at the tail-end of the fog season, the atmosphere of which was recreated to dazzling effect using blue tones and amber tints to mimic the distinctive hues of a London particular.^96^ *The Lodger* was, of course, made at Islington, a building on which thousands of pounds had been spent to keep out exactly the kind of industrial fog that it helped produce during its previous incarnation as a power station. Similarly ironic is the fact that whereas most filmmakers saw fog in the studio as a threat to a production’s finances, *The Runaway Bus* deliberately deployed billowing clouds of artificial fog to hide its miniscule budget: according to its star, Frankie Howerd, fog machines were deployed to obscure the ‘patent phoniness’ of its cheap sets and painted backcloths.^97^

Just as the approaches developed to keep studios fog-free evolved, so too did the techniques used to create artificial fogs, moving from bonfire smoke controlled by ‘a wet blanket or tarpaulin’ via filmmakers applying a filter or a chemical solution to the lens of the camera, to more sophisticated chemical fogs.^98^ As new synthetic fogs were developed, they became increasingly complex and costly. In 1939, the special effects department at Warner Bros. studio in California claimed that ‘more labour and expense are involved in providing fog that will satisfy head cameramen than in any other studio effort’.^99^ It does not seem unreasonable to assume that this was also true in a British production context. Some of this expense was associated with synthetic fog’s tendency to dissipate in the mechanically controlled climate of the sound stage and its tendency to be forced by the higher air-pressure inside the studio out through the same gaps that had previously allowed real fogs in. Indeed, *Motion Picture Studio* carried a humorous, and possibly apocryphal, story about the production of George Fitzmaurice’s *Three Live Ghosts* (1922) at Islington, in which the studio’s air-washing plant proved to be *too* effective during the shooting of a sequence set in a pub: ‘Mr Fitzmaurice’s compliments, and could Major Bell [the studio’s manager] do something to stop that machine. Forty “extras” are smoking their hardest to get the proper bar room atmosphere, and the air-washing fiend is clearing away the smoke as fast as it is produced.’^100^

During the making of BIP’s *Contraband* (1934), three different synthetic fogs were used after the real fog then affecting Elstree was actively encouraged into the studio but found wanting. The first made the set so slippery that actors had some difficulty keeping their feet (steam based synthetic fogs could also condense on the camera lens); the next was created by heating sulphate of ammonia in a frying pan until it ‘sizzled like a sausage but smelt like nothing on earth’; and the last ‘came out of a tube like a Roman Candle’.^101^ According to Edward Carrick, in his 1949 book on film design, ‘The type of fog used varies according to the cameraman and the susceptibility of the stars’; some artificial fogs could make ‘actors with delicate throats choke a little’.^102^

Just as London particulars could be injurious to health, so their cinematic analogues could be similarly unpleasant to work in, with some demonstrating a habit of ‘getting into your lungs and sitting there’.^103^ When *Public Nuisance No. 1* was filmed at Beaconsfield in 1935, the synthetic fog

proved highly irritant to sensitive eyes, noses and throats. Electricians, cameramen, director and scenarist – all suffered but none quite so much as the star [Frances Day], who was completely enveloped in it [and] it was with smarting eyes and an ever-threatening huskiness in her voice that she sang bravely on – at one point even having to endure a close-up standing over a barrel of smoke, the smell of which proved abominable.^104^

Kenneth More remembered that the *ersatz* fog used during the filming of *Scott of the Antarctic* (1948) made the actors cough, which had the unintended consequence of preparing them for the ‘choking fog’ that enveloped Leicester Square and delayed the arrival of John Mills, the film’s lead, when it was shown at the Royal Command performance.^105^

Given that actors’ unions would assert that later glycol-based artificial fogs posed a risk to actors, it is hard to believe that earlier processes, such as a portable device used on location by Rank in the 1950s which synthesised fog by heating diesel to the point of vaporisation, were not also potentially harmful.^106^ Crews might be able to wear masks, as they are reported to have done during the filming of *The Runaway Bus*, but this was not an option for actors.^107^ It is not surprising, then, that the studios sought to create artificial fogs that were less unpleasant to work in, and Twickenham’s property chief, Charles Hasler, was credited by *Kinematograph Weekly* with perfecting an odourless synthetic fog for use in *Scrooge* (1935) that ‘does not choke the technicians who have to work in it’.^108^

Fog’s status as the quintessential London weather event, as a visual and meteorological shorthand for the city, meant that filmmakers outside Britain were keen to replicate it convincingly. German technicians, for example, developed techniques to produce to order pea-soupers ‘materially sufficient to confirm a specific location and atmosphere’.^109^ Indeed, the synthetic fogs developed by German studios were often superior to those produced in Britain, and examples of them being imported for use in British productions include the fog used in Gaumont-British’s *Channel Crossing* (1933) and the ‘special candles’ used by the Rank Organisation in 1950.^110^ A particularly interesting example can be found in the production of *The Amateur Gentleman* (1936), for which cameraman Gunter Krampf ‘insisted on sending to the Continent for the latest thing in fogs,’ which were duly flown in to Elstree.^111^ A German who worked in Britain from 1931, Krampf had returned to Germany in the winter of 1934-5 to shoot *Das Mädchen Johanna*, a film whose strikingly photographed fog likely informed *The Amateur Gentleman*.^112^ James Mason later recalled that Krampf was ‘a perfectionist’ when it came to fog, although for obvious reasons accessing German-made fog was impossible by the time that the pair worked together at Welwyn on *The Night Has Eyes* (1942).^113^

## Conclusion

The impact that fog, and the weather more generally, had on British film production serves to remind us that filmmaking is an activity that occurs in specific places, and that these places work to shape the film industry in myriad ways. It is important that we attempt to imbricate the specificities of place within our histories of the spaces of film production: studios do not exist in isolation, but are located in, and in turn become part of, environments and ecosystems – they both bear the imprint of, and internalise, their interactions with their location. Explorations of the locatedness of the physical infrastructures of filmmaking – and, more generally, of material and popular culture and the industries that support them^114^ – needs to extend beyond the national, the political and the architectural to encompass the geographical, the geological and the meteorological; film production, both on location and in the studio, is subject to forces that do not recognise national boundaries, production schedules, budgets or labour agreements.

The climate is a global system, but one that manifests itself in localised ways. Whilst the impact that the weather had on film producers in Britain relates specifically to the climate prevailing in the UK, filmmakers in other countries have also found that local climatic factors have influenced the places in, processes by, and the equipment with which films are made. For the winter fog in Britain, we might, for example, substitute the monsoon in India, where the annual rainy season continues to inspire seasonal migration.^115^

The film studio constitutes an attempt to deny the climate and introduce a single, standardised internal environment optimised to the needs of film production. However, by focussing on the products of these aclimatic spaces, we risk denying the influence that natural or meteorological factors have had on different film industries; the differing climatic conditions in California, London, Lagos or Mumbai will shape filmmaking practices and infrastructure in different ways – unique problems require unique solutions. Recognising this, and thinking about the relationships between filmmaking and the natural and built environments, might constitute a step in the direction of reintegrating the natural into the (understandably) anthropocentric focus of film studies, and its frequent focus on an industry whose relationship with the real world was defined by its rejection, and subsequent artificial recreation, of natural processes.

For many British filmmakers, fog was productive of an uncanny working environment. That the British film industry was for decades forced to contend with fogs capable of forcing their way *inside* buildings feels more like the premise of a horror film than an attestable historical event. Indeed, the language used to describe the impact of fog on British filmmakers, whether working inside or outside the studio, was often supernatural in tone, with fog described variously as a ‘demon,’ an ‘ogre,’ a ‘fiend’ and the ‘greatest bugbear’ of British producers.^116^ The rude intrusion of the natural world into the spaces of a production sector that sought to exclude it, only to recreate it as simulacrum, might, therefore, constitute the return of the repressed. The most disruptive urban fogs were anthropogenic, products of the exploitation of the natural world by human activity: the film industry – often wasteful, frequently dirty, and a voracious consumer of natural resources – was disrupted by the same industrial pollution for which it was, in part, responsible for creating. The fog was, to a degree, a monster of the film industry’s own making.

## Notes

1 By the 1920s, the worst London fogs were estimated to contain 70 tons of airborne pollutants. *Bioscope - Cleanliness in the Cinema Supplement*, November 13, 1929, ii. ‘Smog’ was a portmanteau coined by Henry Des Voeux in a letter published in *The Times*, December 27, 1904, 11. As the term did not gain widespread usage until the 1950s, I have used the word ‘fog’ unless ‘smog’ was used by a contemporary author.

2 *Kinematograph Weekly*, February 10, 1921, 84.

3 Six studios were interrupted by the Great Fog of December 1952, *Variety*, December 17, 1952, 53. Fog also posed problems for British exhibitors, making travel to the cinema more difficult and entering the auditorium. At the grand opening of the Trocadero, Elephant and Castle, the fog was so thick that ‘the screen was barely visible from the operating box’, *Daily Telegraph*, December 23, 1930, 9.

4 See Kristi McKim, *Cinema as Weather: Stylistic Screens and Atmospheric Change* (London: Routledge, 2013).

5 Recent work on the London fog includes: Peter Brimblecombe, *The Big Smoke: A History of Air Pollution in London since Medieval Times* (Abingdon: Routledge, 1987); Christine L. Corton, *London Fog: The Biography* (Cambridge, MA: Belknap Press, 2015); Lynda Nead, *The Tiger in the Smoke: Art and Culture in Post-War Britain* (New Haven, CT: Yale University Press, 2017).

6 Nead, *Tiger in the Smoke*, 28.

7 Peter Ackroyd, *London: The Biography* (New York: Doubleday, 2000), 432; J. G. Brazell, *London Weather* (London: HMSO, 1968), 111–14.

8 Brian R. Jacobson, *Studios Before the System: Architecture, Technology, and the Emergence of Cinematic Space* (New York: Columbia University Press, 2015), 182.

9 *Architectural Review*, November 1, 1936, 189.

10 *Architects’ Journal*, December 3, 1936, 771.

11 *Kinematograph Weekly – Pinewood Supplement*, October 1, 1936, vii.

12 *Observer*, November 7, 1937, 13.

13 *Annual Report of the Meteorological Committee to the Air Council, 1925–26* (London: HMSO, 1926), 49.

14 Stephen Bottomore, ‘“Weather Cloudy – No Sun”: Filming in Britain for the Edison Company in 1913, from Charles Brabin's diary’, *Film History* 15, no. 4 (2003): 408.

15 *Camera!*, August 21, 1920, 3.

16 *Kinematograph Weekly*, August 4, 1921, v.

17 Leslie Wood, *The Miracle of the Movies* (London: Burke, 1947), 193.

18 *Referee*, April 16, 1911, 11.

19 *Daily Telegraph*, November 18, 1910, 5. Sir Arthur Hill, director of the Royal Botanic Gardens, noted that severe fogs left ‘sooty deposits on the glasshouses that were so sticky that the rain had little effect upon them.’ Just as these deposits reduced light levels to the extent that they had the potential to harm plants, so they could have a deleterious effect on the filmmaking in studios reliant on natural light. *The Times*, October 16, 1936, 11.

20 *Bioscope*, November 24, 1910, 7.

21 *Kinematograph Weekly* – *Kine Technicalities Supplement*, June 2, 1921, xv.

22 *Bioscope*, July 2, 1925, 27.

23 *Kinematograph Weekly* – *Kine Technicalities supplement*, October 19, 1922: iii.

24 *Kinematograph Weekly*, March 22, 1923, 111.

25 *Kinematograph Weekly*, February 10, 1921, 84.

26 *Bioscope*, December 27, 1923, 12.

27 *Kinematograph Weekly*, May 22, 1924, 66. Filming had been scheduled to commence in August 1923, but difficulties in assembling the cast meant that shooting was pushed back into the fog season.

28 *Bioscope*, December 27, 1923, 12. Cecil Hepworth’s *Comin’ Thro’ the Rye* (1923) was another casualty of the winter weather: rain disrupted exterior photography, whilst fog, the ‘principal enemy,’ ‘penetrated the [Walton-on-Thames] studio to impede the taking of interiors’.

29 Some of the costs accruing from bad weather might have been covered by weather insurance. Policies taken out by film producers tended to insure against the interruption of location filming by abnormal weather events; when the shooting of a spectacular sequence of *King of the Damned* (1936) was delayed by a thunderstorm, the estimated £2,000 cost was defrayed by a policy (*Daily Mail*, June 26, 1935, 9). I have found no evidence that British producers could insure against fog.

30 The *Sheffield Daily Telegraph* noted on October 14, 1926 (4) that ‘most of the British cinema companies will cease active operations next month’.

31 Jesse L. Lasky with Don Weldon, *I Blow my Own Horn* (New York: Doubleday, 1957), 132.

32 *Kinematograph Weekly* – *Kine Technicalities supplement*, October 19, 1922, iii.

33 *Bioscope – Service supplement*, November 17, 1927, ix.

34 Jacobson, *Studios Before the System*, 17.

35 *Kinematograph Weekly*, February 24, 1921, xx.

36 *Bioscope*, December 13, 1923, 26. An investigation conducted in late November 1923 found that London fogs often thickened during the morning, peaking around midday day before dissipating during the afternoon. This might have resulted from patterns of domestic coal consumption. J. S. Owens, ‘The London Fogs of November 25–27, 1923’, *Nature* (December 15, 1923): 862–3.

37 *Bioscope*, December 12, 1928, 82.

38 *Kinematograph Weekly*, December 11, 1930, 30.

39 Jennifer Fay, *Inhospitable World: Cinema in the Time of the Anthropocene* (Oxford: Oxford University Press, 2018), 28–9.

40 Duncan Petrie, *Creativity and Constraint in the British Film Industry* (London: Palgrave Macmillan, 1991), 33.

41 *Motion Picture Studio*, March 11, 1922, 12: Production companies ‘automatically shut down when autumn arrives’ anticipating ‘the rains, cloudy skies, short sunless days, and … that terrible ogre which, after all, is the chief cause of their fears – Fog!’

42 The desire to escape the disruptive effects of winter weather led some American filmmakers to establish seasonal outposts in California before relocating permanently. See Brian R. Jacobson, ‘Fantastic Functionality: Studio Architecture and the Visual Rhetoric of Early Hollywood’, *Film History* 26, no. 2 (2014); 55, 71.

43 *Kinematograph Weekly*, December 7, 1922, 51.

44 Rachael Low, *The History of the British Film, vol. 4: 1918–1929* (New York: Routledge, 1999 [1971]), 222.

45 *Bioscope*, April 1, 1926, 42.

46 *Kinematograph Weekly*, January 6, 1921, 165; *Bioscope*: July 14, 1921, 54, and November 18, 1920, 29.

47 Ackroyd, *London*, 433.

48 *Bioscope*, May 4, 1922, 17.

49 Steve Chibnall suggests that although poor British weather might have motivated the production of 28 ‘Britalian’ films after World War Two, the rising cost of British studio space was probably more important. ‘Rome, Open for British Production: The Lost World of ‘Britalian’ Films, 1946–1954’, *Historical Journal of Film, Radio and Television* 33, no. 2 (2013): 235, 247.

50 Baynham Honri, ‘Will G. Barker … His Studios and Techniques’, *British Journal of Photography* (February 16, 1973): 154; Patricia Warren, *British Film Studios: An Illustrated History* (London: B. T. Batsford, 1995), 19. An unrealised 1925 plan to build a new studio in Brighton was developed after the Meteorological Office advised that the town had ‘the best prospect of sunshine and freedom from fogs.’ *Sunday Pictorial*, November 8, 1925, 17.

51 Basil Dean, *Mind’s Eye: An Autobiography, 1927–1972* (London: Hutchinson, 1973), 143; Baynham Honri, ‘The Golden Ages of Ealing Studios’, *British Journal of Photography* (January 30, 1976): 92.

52 *Bioscope*, September 19, 1928, 15.

53 *Bioscope*, December 12, 1928, 100.

54 *Kinematograph Weekly*, August 22, 1935, 17.

55 *Bioscope*, June 16, 1927, 18.

56 *Gloucestershire Echo*, October 21, 1929, 5; *Daily Herald*, October 21, 1929, 1.

57 National Studio plans were much discussed in the mid-1920s. T. A. Welsh, for example, advocated scrapping existing London studios, which he claimed were ‘practically useless in the winter … because of the risk of fog,’ and construct a new facility ‘well outside the area of fog and smoke,’ *Kinematograph Weekly*, July 2, 1925, 41.

58 Wood, *Miracle of the Movies*, 285.

59 *Bioscope*, December 17, 1925, 24.

60 *Kinematograph Weekly*, March 5, 1936, 43. For fog hindering production at Elstree, see *Picturegoer*, January 27, 1934, 7.

61 *Kinematograph Weekly*, January 11, 1934, 111.

62 Laura Vaughn, ‘Making Connections: The Case of Borehamwood’, *Built Environment* 32, no. 3 (2006): 281.

63 *Manchester Guardian*, February 22, 1928, 12.

64 *Scotsman*, August 25, 1928, 8.

65 *New York Times*, September 2, 1928, 86.

66 *Kinematograph Weekly*, January 15, 1920, 119.

67 *Bioscope*, June 18, 1927, 73. See also Warren, *British Film Studios*, 11.

68 *Bioscope*, December 13, 1923, 26.

69 *Kinematograph Weekly*, January 9, 1936, 105.

70 *Bioscope* June 18, 1927, 71.

71 *Kinematograph Weekly*, August 16, 1928, 60. Acoustic insulation installed during the conversion to sound might also have provided increased thermal insulation, thereby decreasing the likelihood of fog forming.

72 *Kinematograph Weekly*, April 5, 1934, 37.

73 *Kinematograph Weekly – Kine Technicalities supplement*, October 19, 1922, iii

74 Mr Gordon Hall Caine, *Hansard: House of Commons*, March 22, 1927, vol 204, col. 281: Mr Gordon Hall Caine.

75 *Kinematograph Weekly – Kine Technicalities supplement*, October 20, 1921, xv.

76 Lasky, *Blow my Own Horn*, 132.

77 *Variety*, May 28, 1920, 2.

78 *Kinematograph Weekly* – *Kine Technicalities supplement*, October 20, 1921, xv.

79 *Moving Picture World*, November 19, 1921, 344.

80 Mr Gordon Hall Caine, *Hansard: House of Commons*, March 22, 1927, vol 204, col. 281.

81 *Moving Picture World*, November 19, 1921, 344.

82 Ibid.

83 Lasky, *Blow my Own Horn*, 132.

84 Details of the system from: *Kinematograph Weekly* – *Kine Technicalities supplement*, October 20, 1921, xv, xiv; *Moving Picture World*, November 19, 1921, 344.

85 Mr Gordon Hall Caine, *Hansard: House of Commons*, March 22, 1927, vol 204, col. 281.

86 Albert Kaufman, general manager of European Productions for Famous Players-Lasky, quoted in *Bioscope*, January 19, 1922, 7; *Kinematograph Weekly*, November 17, 1921, 51.

87 *Bioscope*, June 18, 1927, 76.

88 *Kinematograph Weekly*, December 1, 1927, 82.

89 *Bioscope*, December 31, 1930, 18.

90 *Kinematograph Weekly*, November 1, 1934, 40A.

91 *Kinematograph Weekly*, September 27, 1927, 93. Emphasis added.

92 *Kinematograph Weekly* – *The Films Act of 1938 supplement*, April 1, 1938, xi.

93 Charlotte Brunsdon, *London in Cinema: The Cinematic City since 1945* (London: BFI, 2007), 45.

94 Ackroyd, *London*, 429, 427. For a detailed exploration, see Jesse Oak Taylor, *The Sky of Our Manufacture: The London Fog in British Fiction from Dickens to Woolf* (Charlottesville: University of Virginia Press, 2016).

95 *Picturegoer*, March 5, 1932, 12.

96 Paul Read, ‘Unnatural Colours': An Introduction to Colouring Techniques in Silent Era Movies’, *Film History* 21, no. 1 (2009): 20.

97 Quoted in Corton, *London Fog*, 295.

98 *Kinematograph Weekly – Kine Technicalities supplement*, August 4, 1921, v.

99 *Dundee Courier*, August 12, 1939, 4.

100 *Motion Picture Studio*, December 24, 1921, 10.

101 *Film Weekly*, January 26, 1934, 20; *Brisbane Daily Standard*, March 10, 1934, 8; *Kinematograph Weekly*, January 11, 1934, 111.

102 Edward Carrick, *Designing for Films* (London: Studio Publications, 1949), 108.

103 *Picturegoer*, July 1, 1933, 30.

104 *Bognor Regis Observer*, July 22, 1936, 3.

105 *Daily Telegraph*, November 30, 1948, 1; *Gaumont British News*, December 2, 1948.

106 Karl G. Ruling, ‘Clearing the Air about Fog’, *TCI* 30, no. 4 (April 1996): 45–8; *British Kinematography* 18, no. 3 (September 1950), 85–6.

107 Andy Merriman, *Margaret Rutherford: Dreadnought with Good Manners* (London: Auram Press, 2011), 133–4.

108 *Kinematograph Weekly*, April 4, 1935, 44.

109 Amy Sargeant, ‘Night and Fog and Benighted Ladies’, *Adaptation* 3, no. 1 (2010): 39.

110 *British Kinematography* 18, no. 3 (September 1950): 88.

111 *Kinematograph Weekly*, December 12, 1935, 30.

112 On Krampf’s British work, see Michael Omasta, ‘Famously Unknown: Gunther Krampf’s Work as Cinematographer in British Films’, in *Destination London: German-speaking emigres and British cinema, 1925–1950*, ed. Tim Bergfelder and Christian Cargnelli (Oxford: Berghahn, 2008). Krampf had earlier worked on *Die Büchse der Pandora* (1929), a film constituting part of a Weimar-era ‘sub-genre of London films’ that employed fog in arresting ways. Tim Bergfelder, Sue Harris and Sarah Street, *Film Architecture and the Transnational Imagination: Set Design in 1930s European Cinema* (Amsterdam: Amsterdam University Press, 2007), 139.

113 Quoted Omasta, ‘Famously Unknown,’ 85.

114 How technologies of cultural production respond to localised factors is explored in Michelle Henning, ‘The Worlding of Light and Air: Dufaycolor and Selochrome in the 1930s’, *Visual Culture in Britain* 20, no. 2 (2020).

115 Some Indian film productions, for instance, continue to ‘leave from Mumbai, Calcutta, Hyderabad or Chennai to reach the various targeted Italian regions’ during the annual monsoon season (May to October) when location shooting becomes more difficult. Marco Cucco and Massimo Scaglioni, ‘Shooting Bollywood Abroad: The Outsourcing of Indian Films in Italy’, *Journal of Italian Cinema & Media Studies* 2, no. 3 (2014): 425.

116 *Bioscope*, November 18, 1920, 29; *Motion Picture Studio*, March 11, 1922, 12; *Dublin Evening Telegraph*, December 9, 1920, 2; *Daily Mail*, October 24, 1919, 6.

